# Commentary on: Round Table Discussion: Making Sense of Current Liposuction Technologies

**DOI:** 10.1093/asjof/ojac007

**Published:** 2022-01-31

**Authors:** Al Aly

**Affiliations:** Department of Plastic Surgery, UT Southwestern Medical Center, Dallas, TX, USA

This article^1^ gathers the opinions of experts in liposuction technologies to help the reader make heads or tails of the variety of instruments currently available for use by plastic surgeons. I commend the authors for trying to accomplish this, as it is a very difficult task (Video 1).

Admittedly, I have little to no experience with most of the technologies discussed in this article except for ultrasound (Vaser) and power-assisted liposuction (PAL). This may be perceived as a negative, but I believe this is a positive because I represent a large segment of the plastic surgery population that also does not have experience with these technologies. Thus, my impressions will be especially relevant to at least this segment of the readership.

Unfortunately, reading this article did not aid me in developing a sense of what technology/technologies would help my practice, but it sparked my interest and helped me with how to approach these technologies.

The most important question that I wanted this article to answer for me was “What technologies do the authors prefer for skin tightening?” I had to read this article multiple times, take extensive notes, and organize those notes before I could come up with one general consensus: Renuvion (Apyx Medical, Clearwater, FL) is effective in skin tightening of large surface areas. However, to demonstrate the lack of consistency from one group of authors to another, DiBernardo and Turer indicated that they use PAL or Vaser in combination with Body Tite in 90% of their cases, which does not include Renuvion at all, leaving me confused. 

The second important question that is of interest to me was “What technologies worked best for different areas of the body?” The different author groups again had variable recommendations, which left me with no clear recommendations.

Interestingly, Duncan reports that she uses Renuvion as a tummy tuck alternative to reduce rectus diastasis by theoretically tightening the linea alba. She does not claim that it replaces abdominoplasty plication but that it works when there is a need for less aggressive narrowing of the separation. I find this surprising because in my hands it is difficult to accurately ascertain small nuances of diastasis when examining the abdominal wall through the skin and underlying fat, especially in patients who have moderate to thick panniculi. I believe that it would be difficult for me to judge whether Renuvion had an effect on diastasis, especially because Duncan implies that this should be considered when a relatively small amount of tightening is required. Thus, it seems to me that it would be difficult to physically determine the extent of improvement, unless this was backed by magnetic resonance imaging (MRI) or computed tomography (CT) confirmation. I will concede, however, that it is feasible that Duncan is significantly better at physically determining the extent of rectus diastasis than I am.

One question I ask myself before and after reading this article is “why aren’t these technologies more mainstream?” Three potential answers come to mind:

1. Are these newer technologies only effective in very experienced hands based on the dictum that the success or failure of liposuction is often based on what is proximal to the cannula, in other words, “the skill of the surgeon?” For example, DiBernardo and Turer use laser liposuction for many things and are very happy with its results. In my experience, some of the worst complications that I have seen after liposuction occurred in patients who underwent laser liposuction. Thus, some of these technologies can be very dangerous in inexperienced hands and it is difficult for plastic surgeons who see these complications to contemplate including them in their practices because all they see are difficult problems that resulted from their use.
2. Are these technologies too expensive, especially if it is felt that more than one is required to attain the potential results? Although producing better results, like tightening of the skin, can increase a plastic surgeon’s income, these technologies do not easily show a positive profit to cost ratio.
3. There is a lack of formal education about these technologies, which is often left up to the companies that produce the technologies to educate surgeons. Obviously, these companies, which have hundreds of thousands of dollars in profit potential, are not always the best source of education. So, for someone like me, I have developed a reluctance to purchase these technologies because I feel that the companies have much less interest in how effective their products are, they simply want to make money.

What did I get out of this article? Reading the article very carefully made me realize what questions I should be asking when evaluating these technologies. Some of the questions were instigated by the article, while others crystallized from thinking about the subject in general. I would like to share some of these questions with you:

1. Which technologies cause significant skin tightening?
2. How much skin tightening is discernible?
3. How do you measure that tightening?
4. How long does it last?
5. How safe are these technologies and can you combine them?

Lastly, I believe that this is article should be read and used as a springboard for any plastic surgeon contemplating acquiring liposuction technologies.
